# Intestinal occlusion by gynecological cancers treated by percutaneous endoscopic gastrostomy and lanreotide: an Aviano National Cancer Institute experience

**DOI:** 10.1007/s00520-020-05745-x

**Published:** 2020-09-10

**Authors:** Martina Budel, Luca Martella, Laura Zambon, Isabella Morson, Giorgio Giorda, Renato Cannizzaro

**Affiliations:** 1 Oncological Gastroenterology, Centro di Riferimento Oncologico di Aviano (CRO) IRCCS, Aviano, Italy; 2 Gynecological Surgical Oncology, Centro di Riferimento Oncologico di Aviano (CRO) IRCCS, Aviano, Italy; 3 S.O.C. Gastroenterologia Oncologica Sperimentale, Centro di Riferimento Oncologico, Istituto Nazionale Tumori, IRCCS, Via Franco Gallini 2, 33081 Aviano, PN Italy

**Keywords:** Gynecological cancers, Percutaneous endoscopic gastrostomy, Lanreotide

## Abstract

The Commentary reports on our experience in Centro di Riferimento Oncologico IRCCS Aviano about the integrated and combined treatment with percutaneous endoscopic gastrostomy and lanreotide in patients with bowel obstructions by ovarian cancer and peritoneal carcinomatosis. We treated patients with gynecological cancers and bowel obstruction with percutaneous endoscopic gastrostomy and, when patients were partially responsive, with lanreotide. We registered a constant overall benefit for the quality of life and for the control of symptoms, which is very important especially during the home care follow-up of terminal patients.

Ovarian cancer is the eighth most common type of cancer in the world and it has the lowest survival rate of all gynecological cancers. In 2020, there is an estimation of about 21.750 new cases diagnosed per year and about 13,940 ovarian cancer deaths in the USA [1]. In many cases at the time of diagnosis, it is already at an advanced stage. Malignant bowel obstruction is a common and distressing complication of advanced gynecological cancer and peritoneal carcinomatosis. The obstruction gives rise to a vicious cycle of increased intestinal secretion and fluid accumulation with the resultant damage to the intestinal epithelium causing an inflammatory response [2]. The symptoms are principally nausea, vomiting, and abdominal pain: all these symptoms are debilitating for the patient and difficult to manage at home [3]. Management of obstruction due to advanced cancer is likely to require intravenous hydration and parenteral nutrition alongside pharmaceutical interventions and the use of nasogastric tubes; however, these procedures are distressing for the patients. For this reason, the treatment of the terminal patients to improve and ensure a good quality of life is the primary goal of palliative care [4].

Almost all the patients with gynecological cancer have undergone multiple surgical treatments as well as a number of cycles of chemo- or radiotherapy [5]. The patients normally have a poor performance status, thus making surgical treatment a poor option due to the high mortality and morbidity rate [6, 7].

To date, gastrointestinal decompression through a nasogastric tube is the first-line procedure in patients with disseminated peritoneal carcinomatosis and small bowel obstruction, but this treatment is poorly tolerated and it has many side effects, involving wing necrosis, laryngeal disorders, esophageal-gastric lesions, or aspiration pneumonia.

Percutaneous endoscopic gastrostomy (PEG) is a simple method for achieving nonsurgical gastric decompression in patients suffering from metastatic abdominal tumors and upper gastrointestinal tract obstruction [6].

Decompressive PEG is an endoscopic medical procedure in which a tube is passed into the patient’s stomach through the abdominal wall [9]. PEG is relatively easy to use and allows obstructive symptoms to be resolved in the majority of patients. Special medical skills are not required and the patient may be easily managed at home together with support therapy and pain management. Once PEG has been performed, it is possible to take fluids and semi-liquid foods, offering the patient a chance to taste flavors [12].

PEG offers advantages over the commonly used nasogastric tube and is not as invasive as the traditional gastrostomies, thus reducing the risk related to the classical surgical procedure that is performed only in few selected cases [10].

From our past experience, with the study of Zucchi et al., we showed that, although these patients often have ascites, advanced carcinomatosis and multiple gastrointestinal treatments, PEG, and percutaneous endoscopic jejunostomy (PEJ) were positioned with few side effects. Results showed an improvement of symptomatology, assessed for the first time through a subjective score, Symptom Distress Score (SDS), proving that the PEG treatment is appropriate in terminal patients unfit for surgery [8].

In terminal patients with gynecological cancers, the placement of decompressive PEG can be associated with analogues of somatostatin, a molecule that inhibits the secretion of different hormones, such as insulin and gastrointestinal hormones. In vivo, somatostatin has a biological half-life of 2–3 min, allowing only intravenous infusion [10].

The development of analogues of somatostatin, such as lanreotide and octreotide with long-acting formulations, administered at 4- or 2-week intervals, respectively, minimizes the discomfort of the patient. These drugs also modulate gastrointestinal function by reducing gastric and intestinal secretions and slowing the intestinal motility [11]. In particular, we used lanreotide, a more stable molecule with longer half-life than somatostatin, 8 h versus 2–3 min, and few side effects, such as asymptomatic gallbladder microlithiasis, initial diarrhea, and mild abdominal pain [7]. Moreover, lanreotide seems to be easier to handle than other analogues: the plasma levels tend to decrease slowly, above 1 ng/ml for 14 days after the administration, so it can be administered at 10–14-day intervals by the general practitioner during the home care of the patients.

We studied 27 patients with gynecological cancer, peritoneal carcinomatosis, and small bowel obstruction, treated at the Department of Gynecological Surgical Oncology of the Centro di Riferimento Oncologico IRCCS Aviano, Italy.

The majority of them had surgical treatment and received also two or three cycles of chemotherapy. They were hospitalized with clinical and radiological diagnosis of complete intestinal obstruction.

PEG was performed when there were no other solutions than palliative derivation. When symptoms worsened, we introduced lanreotide, at the starting dose of 30 mg.

Most patients received at least two doses of lanreotide at 14-day intervals. After one or two administrations, we observed reduction of nausea in 68.8% of them, of vomiting in 50.7% of cases, and of distension or abdominal pain in 42.8% of cases (Fig. 1). In particular, based on the SDS questionnaire, in 80.5% of patients treated with 2 infusions of the PEG procedure and lanreotide, we recorded a consistent improvement in the overall quality of life, especially for them and their relatives during home care.

**Fig. 1:**
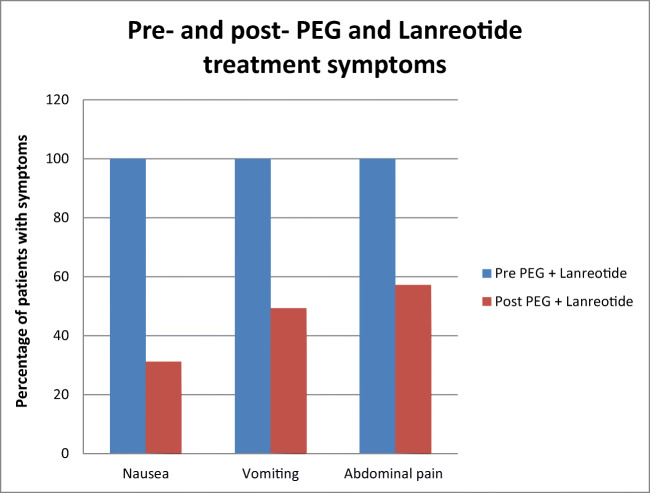
Percentage of patients with pre- and post-PEG + lanreotide treatment symptoms (nausea, vomiting, and abdominal pain)

The positioning of PEG facilitates the home care follow-up in those patients with neoplastic small bowel obstruction, and lanreotide is the drug of choice to be associated in the control of symptoms.

In conclusion, based on our experience, the association of PEG and lanreotide is a valid tool for the palliation of malignant inoperable bowel obstruction in gynecological cancer patients and shows a better cost-benefit ratio than other options. During the home care follow-up, this treatment increases the chances of avoiding hospitalization and permitting, potentially, a better quality of life.
